# Campylobacter Fetus Meningitis in Adults: Report of 2 Cases and Review of the Literature

**DOI:** 10.1097/MD.0000000000002858

**Published:** 2016-03-03

**Authors:** Anusha van Samkar, Matthijs C. Brouwer, Arie van der Ende, Diederik van de Beek

**Affiliations:** From the Department of Neurology (AVS, MCB, DVDB); Department of Medical Microbiology (AVDE), Academic Medical Center, Center of Infection and Immunity Amsterdam (CINIMA); and Netherlands Reference Laboratory for Bacterial Meningitis (AVDE), Academic Medical Center, Amsterdam, The Netherlands.

## Abstract

The zoonotic pathogen *Campylobacter fetus* is a rare cause of bacterial meningitis. Little is known about the clinical characteristics, predisposing factors and outcome of *C fetus* meningitis in adults.

We report cases of *C fetus* meningitis in a nationwide cohort study of adult bacterial meningitis patients in the Netherlands and performed a review of the literature.

Two patients with *C fetus* meningitis were identified from January 2006 through May 2015. The calculated annual incidence was 0.02 per million adults. Combined with the literature, we identified 22 patients with a median age of 48 years. An immunocompromised state was present in 16 patients (73%), mostly due to alcoholism (41%) and diabetes mellitus (27%). The source of infection was identified in 13 out of 19 patients (68%), consisting of regular contact with domestic animals in 5 and working on a farm in 4. Recurrent fever and illness was reported in 4 patients (18%), requiring prolonged antibiotic treatment. Two patients died (9%) and 3 survivors (15%) had neurological sequelae.

*C fetus* is a rare cause of bacterial meningitis and is associated with an immunocompromised state. Based on the apparent slow clinical response seen in this limited number of cases, the authors of this study recommend a prolonged course of antimicrobial therapy when *C fetus* is identified as a causative agent of bacterial meningitis. Cases appeared to do best with carbapenem therapy.

## Abstract 2

Supplemental Digital Content is available in the text

## INTRODUCTION

Bacterial meningitis is a severe infectious disease requiring prompt antibiotic treatment. Most cases are caused by *Neisseria meningitidis* and *Streptococcus pneumoniae*, which are both part of the commensal nasopharyngeal flora in humans.^1^ Bacterial meningitis is rarely caused by bacteria having their natural reservoir in animals. One of these so-called zoonotic pathogens is *Campylobacter fetus* (formerly *Vibrio fetus, Spirillum serpens*), which is part of the commensal flora in the gastro-intestinal tracts of sheep and cattle.^2^*C fetus* meningitis occurs worldwide, but little is known about its clinical characteristics, predisposing factors and outcome. We report 2 cases of *C fetus* meningitis from a nationwide cohort of bacterial meningitis patients in the Netherlands. Additionally, we performed a review of the literature on *C fetus* meningitis.

## METHODS

We included patients with community-acquired bacterial meningitis in a nationwide prospective cohort study in the Netherlands between January 2006 and May 2015. Methods have been described previously.^1^ Patients were listed in the database of the Netherlands Reference Laboratory for Bacterial Meningitis (NRLBM), which receives >90% of the cerebrospinal fluid (CSF) isolates of all adult patients (>16 years) with CSF culture confirmed bacterial meningitis. The NRLBM provided daily updates of the hospitals where the patients were admitted and the patients’ physicians, who were subsequently contacted. Physicians could also include patients without report of the NRLBM. Written informed consent was obtained from all patients or their legally authorized representatives. The study was approved by the medical ethical review board of the Academic Medical Center, Amsterdam, The Netherlands.

From the cohort, we selected patients with *C fetus* meningitis. Additional information on risk factors was retrospectively collected from the discharge letters. Patients were considered immunocompromised if they had cancer, diabetes mellitus, alcoholism, asplenia, HIV-infection, or use of immunosuppressive medication.^1^

Individual predictive factors in the cerebrospinal fluid were defined as follows: a glucose level of <34 mg/dL (1.9 mmol/L), a ratio of CSF glucose to blood glucose of <0.23, a protein level of >220 mg/dL, or a leukocyte count of >2000/mL (Spanos criteria).^3^

### Review of the Literature

We performed a literature search using the search terms “*Campylobacter fetus* AND meningitis,” “*Vibrio fetus* AND meningitis,” and “*Spirillum serpens* AND meningitis.” Studies written in English, German, French, Dutch, Spanish, Italian, and Portuguese were included. Articles describing animals and articles describing children were excluded. We also excluded articles in which no subanalysis for *C fetus* meningitis cases was performed, or when no clinical characteristics were described. Additional studies were identified by cross-checking references.

In a meta-analysis of clinical data we systematically scored clinical presentation, predisposing factors, ancillary investigations, and outcome. Differences between groups were calculated by means of Fisher's Exact Test.

## RESULTS

### Case Reports in Prospective Nationwide Cohort Study

Two patients with *C fetus* meningitis were identified in our nationwide cohort consisting of 1732 patients (0.1%). The calculated annual incidence of *C fetus* meningitis in the Netherlands was 0.02 per 1,000,000 adults.

## CASE 1

A 23-year-old woman presented at the emergency department with fever, headache, and earache since 4 weeks and severe neck pain since 3 days. She was previously healthy and had been in regular contact with horses, dogs, rabbits, and guinea pigs. Physical examination showed fever and neck stiffness but no other abnormalities. Blood laboratory examination was normal. CSF examination was consistent with meningitis (Table 1) and the patient was treated with amoxicillin, ceftriaxone, and acyclovir. Cultures became positive for *C fetus* subspecies *fetus* after 9 days. Amoxicillin and acyclovir were discontinued and ceftriaxone was continued for 2 weeks. The patient was discharged, but mild vertigo and a decreased sense of smell remained. One week after discharge, the patient presented with a subfebrile temperature (38–38.5°C) and headache. Repeated CSF examination was consistent with meningitis (Table 1). Despite prolonged treatment with meropenem, the patient's complaints lasted for a total of 4 weeks. Extensive ancillary investigations did not reveal any underlying illness. The patient was not able to resume her studies due to persisting fatigue and cognitive defects.

**TABLE 1:**
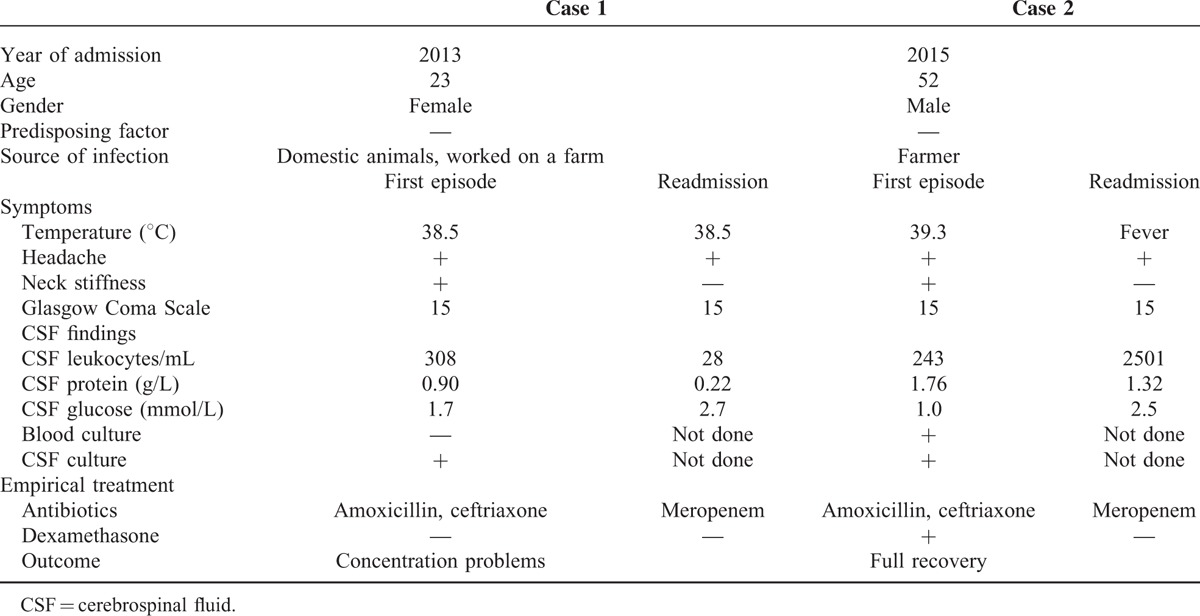
Clinical Characteristics, Etiology, and Clinical Outcome of Cases of *Campylobacter fetus* Meningitis in Our Cohort

## CASE 2

A 52-year-old previously healthy farmer presented at the emergency department with headache and fever since 10 days and a stiff neck since 2 days. Physical examination showed fever and neck stiffness. Blood laboratory examination showed 11.9 × 10^9^ leukocytes/L and a C-reactive protein of 206 mg/L. CSF examination was consistent with meningitis (Table 1). The patient was treated with ceftriaxone and amoxicillin for 2 weeks and received adjunctive dexamethasone 10 mg 4 times a day for 4 days. CSF and blood cultures were positive for *C fetus* subspecies *fetus*. The patient was discharged in good clinical condition, but after a week, he came back to the hospital because of recurrent headache and fever. Physical examination showed fever but no other abnormalities. Blood laboratory examination showed 10.8 × 10^9^ leukocytes/L, and CSF examination was consistent with bacterial meningitis (Table 1). CSF cultures were not repeated. The patient was treated with meropenem for 3 weeks and fully recovered.

### Review of the Literature

**FIGURE 1:**
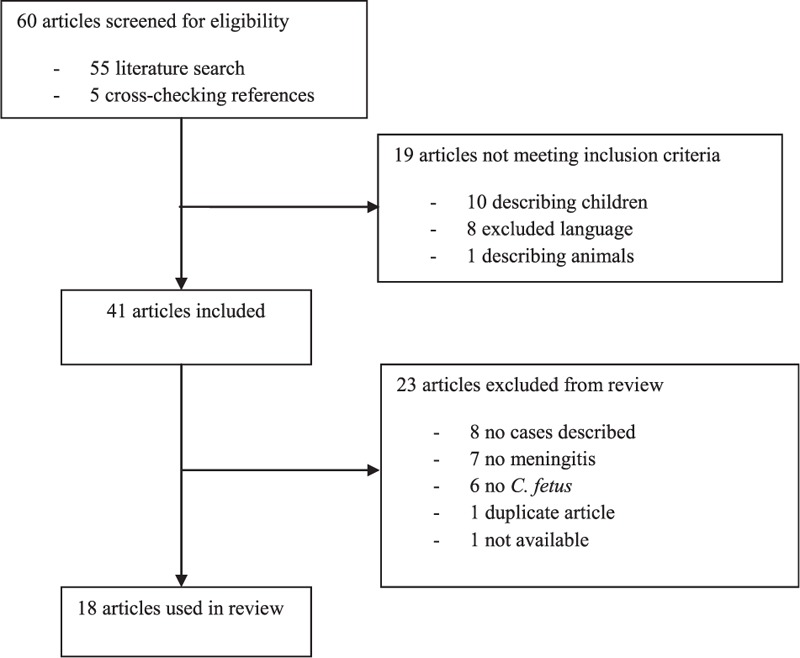
Flowchart review of the literature *C fetus* meningitis. *C fetus = Campylobacter fetus.*

We identified a total of 18 relevant articles published between 1960 and 2013 (Figure 1) (Supplementary Table 1).^4–21^ Combined with our cases, 22 adult patients with *C fetus* meningitis were identified (Table 2) with a median age of 48 years (range 23–84 years). Sixteen patients were men (73%). An immunocompromised state was present in 16 out of 22 patients (73%, 95% CI 54–92%) and consisted of alcoholism in 9 patients, diabetes mellitus in 6, use of immunosuppressive medication in 2, and leukemia and asplenia in 1 patient each. The source of infection was identified in 13 out of 19 patients (68%, 95% CI 47–89%) and consisted of frequent contact with domestic animals in 5 patients (38%, 95% CI 12–64%), working on a farm in 4 (31%, 95% CI 10–52%), frequent contact with rats in 3, consuming raw meat in 2, and working in an abattoir and chewing khat in an animal sanctuary in 1 patient each (Table 1). One patient cared for sick animals before developing meningitis.^20^ Both an immunocompromised state and an identified source of infection were present in 7 out of 19 patients (37%, 95% CI 15–59%).

**TABLE 2:**
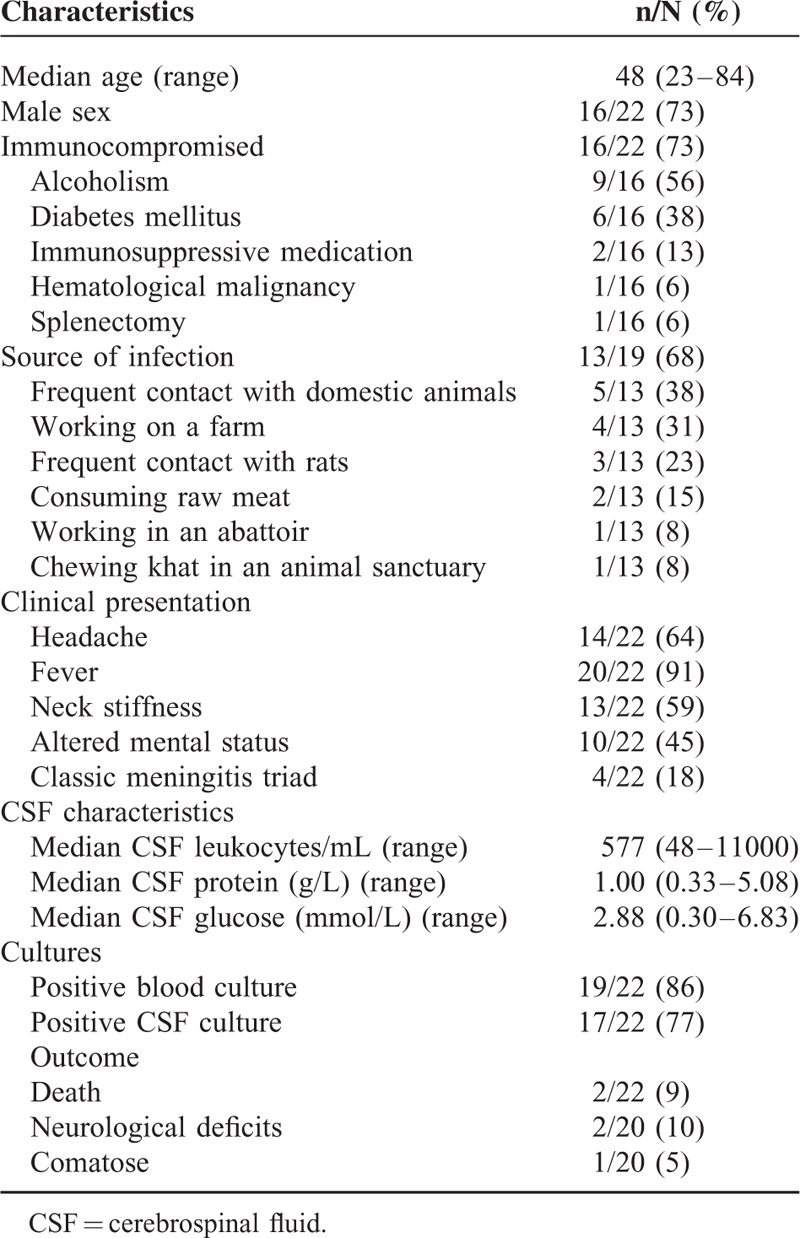
Clinical Characteristics, Etiology, and Clinical Outcome of Cases of *Campylobacter fetus* Meningitis in Adults, Including Our 2 Patients

Presenting symptoms were reported in all 22 patients and consisted of headache in 14 (64%, 95% CI 44–84%), fever in 20 (91%, 95% CI 79–100%), neck stiffness in 13 (59%, 95% CI 38–80%), and an altered consciousness in 10 patients (45%, 95% CI 24–66%). The classic triad of fever, neck stiffness, and an altered consciousness was present in 4 patients (18%, 95% CI 2–34%). At least 2 of the 4 symptoms of headache, fever, neck stiffness, and an altered consciousness were present in all patients. There was no association between the presence of fever and an immunocompromised state (*P* = 0.48).

The results of blood investigations were reported in 15 patients. The median leukocyte count was 12.2 × 10^9^/L (range 5.4–29.3 × 10^9^). The blood leukocyte count was considered normal (range 4.0–10.0 × 10^9^/L) in 4 patients.

CSF examinations were performed in all patients; CSF was abnormal in all (Table 2, Supplementary Table 1). Individual CSF predictive factors were present in 10 out of 19 patients (53%, 95% CI 31–75%), mostly due to a decreased CSF glucose (6 patients). The CSF leukocyte count was <1000 per mL in 11 patients (52%, 95% CI 31–73%), ranging from 48 to 11,000 leukocytes per mL. There was no association between a CSF leukocyte count of <1000 per mL and an immunocompromised state (*P* = 1.00) or alcoholism (*P* = 0.66).

CSF cultures were positive in 17 out of 22 patients (77%, 95% CI 59–95%); in 5 patients, CSF cultures were negative, whereas blood cultures were positive (23%, 95% CI 5–41%). Blood cultures were positive in 19 out of 22 patients (86%, 95% CI 71–100%). Both CSF and blood cultures were positive in 14 out of 22 patients (64%, 95% CI 44–84%). *C fetus* subspecies *fetus* was the causative organism in all cases.

Antibiotic treatment was highly diverse and the primary antibiotic treatment mainly consisted of beta-lactam antibiotics, such as penicillin (between 1960 and 1970), amoxicillin, ampicillin, and ceftriaxone (from 1985 onwards). In 11 patients (50%, 95% CI 29–71%), the antibiotic treatment was altered after the cultures became positive for *C fetus*. The duration of antibiotic treatment was reported in 9 patients: 7 patients were treated for 4 weeks, 1 patient for 5 weeks (case 2), and 1 patient for 6 weeks (case 1).

Outcome was reported in all 22 patients: 2 patients died (9%, 95% CI 0–21%).^9,16^ Three out of 20 survivors (15%, 95% CI 0–31%) had an unfavorable outcome: 1 patient remained comatose, 1 patient had a persisting hemiparesis, and 1 patient had persisting fatigue and concentration problems (case 1). There was no association between any cause of an immunocompromised state and unfavorable outcome (*P* = 0.59).

Including our 2 patients, 4 out of 22 patients (18%, 95% CI 2–34%) had persisting or recurrent fever and headache, for which they were readmitted to the hospital for antibiotic treatment.^8,18^ In 1 patient, the isolated *C fetus* strain was resistant to the prior administered antibiotics (penicillin), and repeated blood and CSF cultures remained positive until another antibiotic agent (tetracycline) was administered.^18^ However, in the other 3 patients, the *C fetus* isolate was susceptible to the antibiotics administered during the first admission (ceftriaxone and amoxicillin in 2 and cefotaxime and vancomycin in 1). After a 3-week treatment with meropenem (2 cases) and ofloxacin and gentamicin (1 case), fever and headache disappeared. In 2 of these 3 patients (case 1 and case 2), no new cultures were performed before this treatment, and in the other patient, new CSF and blood cultures remained negative.^8^

## DISCUSSION

Meningitis caused by *C fetus* is a rare disease, which is associated with an immunocompromised state. Nine patients diagnosed with *C fetus* meningitis had a previous history of alcoholism, and 5 patients had diabetes mellitus. Alcoholism and diabetes mellitus are both risk factors for bacterial meningitis^22^ and have been associated with a high rate of unfavorable outcome.^23,24^ Cancer has been reported to be a risk factor for *C fetus* bacteremia,^25^ but was only present in 1 patient with *C fetus* meningitis.

Although *C fetus* is a zoonotic pathogen, contact with animals or animal products could only be identified in 68% of patients with *C fetus* meningitis. In most patients in whom a source of infection was identified, frequent contact with domestic animals was reported to be the source of infection (38%). However, ∼164 million American households have domestic animals,^26^ implying that the risk at developing *C fetus* meningitis after frequent domestic animal contact is very low.

CSF abnormalities were present in all patients with *C fetus* meningitis. However, only 53% of the cases had at least 1 individual CSF predictor for bacterial meningitis,^3^ as compared to 88% of the patients with community-acquired bacterial meningitis in a large prospective cohort study.^27^ Furthermore, CSF cultures were negative in 23% of the *C fetus* meningitis cases, whereas blood cultures were positive. As blood cultures were positive in 86% of all cases, they can therefore be useful to confirm the diagnosis of *C fetus* meningitis in the case of CSF abnormalities and a negative CSF culture. When *C fetus* meningitis is suspected but cultures remain negative, PCR targeting 16S rRNA encoding gene sequencing followed by sequencing of the PCR product may provide the diagnosis.^28^

*C fetus* has been described to be resistant to several antimicrobial agents. In a multicenter study of 25 isolates of *C fetus* ssp. *fetus* recovered from blood and synovial fluid samples, a significant proportion of isolates was interpreted as intermediate or resistant to ampicillin (12%), cefotaxime (80%), and erythromycin (100%).^29^ Several case reports describe human *C fetus* isolates resistant to ceftriaxone,^30^ cefotaxime,^4,7,8^ and penicillin.^8,12^ In *C fetus,* the genes tet(44) and ant(6)-lb have been associated with resistance to tetracycline, minocycline, and streptomycin.^31^ Other genes may play a role in reduced susceptibility of *C fetus* for antimicrobial agents which are commonly used for the treatment of bacterial meningitis, such as ceftriaxone.^32^ In our study, 4 patients were known to be readmitted to the hospital because of persisting fever and CSF abnormalities, and received prolonged treatment with antibiotics, although the *C fetus* isolate was sensitive to the primarily received antibiotics in 3 of these cases. There might even be some cases where the patients might have persisting or recurrent fever but not readmitted to the hospital for treatment. Relapsing and persisting infection have also been reported in other manifestations of *C fetus*.^33^ This is interesting and suggests inconsistency between the in vivo and in vitro susceptibility of *C fetus*. However, as repeated cultures remained negative in most cases, it is also possible that the recurrent clinical parameters are a postinfectious syndrome or inflammatory response. Nevertheless, cases appeared to do best with carbapenem therapy. Based on the apparent slow clinical response seen in this limited number of cases, the authors of this study recommend a prolonged course of antimicrobial therapy when *C fetus* is identified as a causative agent of bacterial meningitis.^28^

Our study had several limitations. First, only patients with a positive CSF culture were included. In our literature review, 23% of the patients had a negative CSF culture, which means we could have missed cases of *C fetus* meningitis. Second, patients may not have undergone a lumbar puncture due to space-occupying lesions on cranial CT or coagulation problems. Furthermore, we did not include neonates with *C fetus* meningitis, as predisposing factors, etiology, and clinical characteristics in neonates are not comparable to those in adults. Also, specific characteristics of interest were not always available in the retrieved case-reports included in our meta-analysis. Therefore, we reported the number of patients in who the specific characteristic was known.

Finally, the recommendations that can be made are limited by small numbers of affected patients.

In conclusion, *C fetus* is a rare cause of bacterial meningitis and is associated with an immunocompromised state. Based on the apparent slow clinical response seen in this limited number of cases, the authors of this study recommend a prolonged course of antimicrobial therapy when *C fetus* is identified as the causative agent of bacterial meningitis. Cases appeared to do best with carbapenem therapy.

## Supplementary Material

Supplemental Digital Content
