# Supplementary material for: Campylobacter Fetus Meningitis in Adults: Report of 2 Cases and Review of the Literature
Source: Medicine (Baltimore). 2016 Mar 3;95(8):e2858. doi: 10.1097/MD.0000000000002858 (PMC4779013; doi:10.1097/MD.0000000000002858)
Supplement: Supplemental Digital Content [file medi-95-e2858-s001.docx]

**Supplementary table 1**. Cases of *Campylobacter fetus* meningitis reported in the literature, combined with our patients

| Study | Age | Gender | Predisposing factor | Source of infection | Symptoms | CSF leukocytes/mL | Blood culture | CSF culture | Outcome |
| --- | --- | --- | --- | --- | --- | --- | --- | --- | --- |
| Suy^4^ | 75 | Male | DM | Raw sheep liver ingestion | 2;4 | 1430 | + | + | Full recovery |
| Martinez^5^ | 28 | Male | No | Khat chewing | 1;2 | 170 | + | - | Full recovery |
| Umehara^6^ | 40 | Male | Prednisolone | Unknown | 1;2 | 115 | + | + | Full recovery |
| Herve^7^ | 71 | Male | DM | Unknown | 3;4 | 11100 | + | + | Full recovery |
| Dronda^8^ | 47 | Male | Alcoholism | Dogs, cats | 1;2;3 | 300 | + | - | Full recovery* |
| Wilhelm^9^ | 84 | Male | Alcoholism | N.R. | 2;4 | 577 | + | + | Death |
| Kato^10^ | 55 | Male | Alcoholism, DM | Unknown | 1;2;3;4 | 400 | + | + | Full recovery |
| Clavelou^11^ | 39 | Female | Alcoholism | N.R. | 2;3 | 1800 | + | + | Full recovery |
| Clavelou^11^ | 36 | Male | Alcoholism | N.R. | 2;4 | 154 | + | - | Full recovery |
| Rao^12^ | 47 | Male | DM, ISM | Raw calve liver ingestion | 1;2 | 48 | + | + | Full recovery |
| Malbrunot^13^ | 38 | Male | No | Cats | 2;3 | 2040 | - | + | Full recovery |
| Gubina^14^ | 46 | Male | Alcoholism | Farmer, domestic animals | 1;2 | N.R. | + | - | Full recovery |
| Gubina^14^ | 40 | Male | No | Domestic animals | 1;2 | 2821 | + | + | Full recovery |
| Gunderson^15^ | 53 | Male | Alcoholism | Unknown | 1;2;3;4 | 7250 | + | + | Comatose |
| Reyman^16^ | 69 | Female | DM, splenectomy | Unknown | 2;4 | 1230 | + | + | Death |
| Stille^17^ | 50 | Male | DM | Abattoir worker | 1;2;3 | 3436 | - | + | Full recovery |
| Collins^18^ | 55 | Male | Leukaemia | Rats at work place | 1;2;3 | 330 | + | + | Full recovery* |
| Robin^19^ | 47 | Female | Alcoholism | Unknown | 1;2;3;4 | 2128 | + | + | Full recovery |
| Killam^20^ | 48 | Female | No | Farmer, cared for sick calves | 3;4 | 1399 | + | - | Hemiparesis |
| Edwards^21^ | 50 | Female | Alcoholism | Lived in rat-infested neighbourhood | 1;2;3;4 | 100 | + | + | Full recovery |
| This study | 23 | Female | No | Farmer, domestic animals | 1;2;3 | 308 | - | + | Concentration problems* |
| This study | 52 | Male | No | Farmer | 1;2;3 | 243 | + | + | Full recovery* |

*These patients were readmitted due to persisting symptoms. Abbreviations: N.R: Not Reported; CSF: cerebrospinal fluid, DM: Diabetes Mellitus. ISM: immunosuppressive medication. Symptoms: 1 headache; 2 fever; 3 neck stiffness; 4 altered consciousness.
